# The role of inflammation and cortisol in the relationship between social cognition abilities and later emotional or behavioural problems: evidence from a UK birth cohort

**DOI:** 10.1192/j.eurpsy.2022.928

**Published:** 2022-09-01

**Authors:** D. Ji, S. Papachristou, M. Francesconi

**Affiliations:** University College London, Psychology And Human Development, London, United Kingdom

**Keywords:** social cognition, general population, Cortisol, prospective cohort study

## Abstract

**Introduction:**

Deficits in social cognition have been associated with the onset of emotional and behavioural problems, but the biological mechanisms underlying this relationship remain unclear.

**Objectives:**

This study examined whether diurnal cortisol patterns, systemic inflammation, or both, explained the association between social cognition difficulties and subsequent emotional and behavioural symptoms.

**Methods:**

The sample consisted of 714 individuals from the Avon Longitudinal Study of Parents and Children (ALSPAC) with valid data on cortisol measures (age 15 years) and emotional or behavioural problems (age 17 years). Social cognition abilities were measured at 8, 11, and 14 years old. Inflammation was measured using serum levels of interleukin 6 (IL-6, age 9 years) and C-reactive protein (CRP, age 9 and 16 years). Bayesian structural equation modelling was used to investigate the mediating effect of cortisol or inflammation on the association between social cognitive difficulties and emotional or behavioural problems.

**Results:**

Children with social cognition difficulties were associated with later emotional and behavioural problems. Flattened diurnal cortisol slope was associated with the hyperactivity/inattention problem two years later. Mediation analyses revealed that lower morning cortisol significantly mediated the associations between social communication difficulties at 8 years with hyperactivity/inattention and conduct problems in adolescence, with the adjustment of inflammation and all covariates. Systemic inflammation was not related to social cognitive difficulties or future emotional and behavioural problems.

**Conclusions:**

The finding suggests that social cognition is related to cortisol activities longitudinally. It also expands the evidence that adolescents with behavioural problems are characterised by hypoactivity of the hypothalamic-pituitary-adrenal (HPA) axis.

**Disclosure:**

No significant relationships.

